# 

**Published:** 1840-06

**Authors:** 


### CONTENTS

OF NO. VI.

### ORIGINAL COMMUNICATIONS.

### ESSAYS AND CASES.

ART. I.—An Essay on the Fevers which prevailed in Natchez in 1837, '8 and '9. By SAMUEL HOGG, M. D. - - - 401
ART. II.—Cases of Fracture of the Skull, presenting some singular phenomena. By SAMUEL G. DICKINSON, M. D. - - 424
ART. III.—Proofs of the Health-preserving properties of the Jussieua Grandiflora. By SAMUEL A. CARTWRIGHT, M. D. - 428
ART. IV.—A Case of Purpura Hemorrhagica successfully treated. By WILLIAM SEARCY, M. D. - - - - - 452
ART. V.—A Case of Concussion of the Brain, with remarkable recovery. By J. L. BURTT, M. D. - - - - - 455

### REVIEWS.

ART. VI.—Researches Anatomical and Physiological. By JOHN DAVY, M. D. Electricity of the Torpedo. - - - 457

### SELECTIONS FROM AMERICAN AND FOREIGN JOURNALS.

On Creosote as a remedy for Deafness. - - - - - - - 469

Abernethy as a Lecturer. - - - - - - - - - - 471
Abernethy at the Last. - - - - - - - - - - - 471
State of the Faculty and of Physic in Algiers. - - - - 472

DOMESTIC INTELLIGENCE.

On the new Vegetable Alkalies, Veratria, Delphinia, & Aconitine in Neuralgia, and toothache. By SAMUEL A. CARTWRIGHT, M. D. - 473
Medical Graduates in the U. S. - - - - - - - - 477
A Case of forcible extraction of the Uterus. - - - - - 478
A Case of Artificial Anus cured. - - - - - - - - 481
Permanent Cure of Hernia. - - - - - - - - - 481
Treatment of Dysmenorrhœa with the Pessary. - - - - 482
Fevers of Alabama. - - - - - - - - - - - 482
Surgical Anatomy. By Prof. GROSS. - - - - - - 484
Boston Medical and Surgical Journal. - - - - - - 484

